# Supplementary material for: Evolutionary persistence and divergence of the tdk killer meiotic driver family
Source: bioRxiv. 2025 Dec 29:2025.12.28.696746. Preprint. [Version 1] doi: 10.64898/2025.12.28.696746 (PMC12930445; doi:10.64898/2025.12.28.696746)
Supplement: 1 [file NIHPP2025.12.28.696746V1-supplement-1.pdf]

bioRxiv preprint doi: https://doi.org/10.64898/2025.12.28.696746; this version posted December 29, 2025. The copyright holder for this preprint (which was not certified by peer review) is the author/funder, who has granted bioRxiv a license to display the preprint in perpetuity. It is made available under aCC-BY 4.0 International license.

a

Previous systematic ID	Updated systematic ID	Gene symbol	Pairwise identity with Tdk1
SPCC613.11c	*SPCC613.11c*	*tdk11*	28.7%
SPOG_05058	*SZCRYO_07103*	*tdk201*	22.4%
SPOG_05700	*SZCRYO_07146*	*tdk203*	27.3%
SPOG_03626	*SZCRYO_07162*	*tdk205*	24.8%
SPOG_03615	*SZCRYO_09024*	*tdk207**	18.2%
SPOG_02247	*SZCRYO_02247*	*tdk210*	27.7%
SPOG_03585	*SZCRYO_07256*	*tdk217*	23.4%
SPOG_01635	*SZCRYO_07288*	*tdk220*	21.3%
SOMG_00017	*SOMG_00017*	*tdk301*	24.4%
SOMG_00021	*SOMG_00021*	*tdk303*	24.7%
SOMG_00451	*SOMG_00451*	*tdk304*	23.5%
SOMG_02498	*SOMG_02498*	*tdk308*	19.6%
SOMG_04991	*SOMG_04991*	*tdk314*	23.0%
SOCG_01873	*SZOCTO_07189*	*tdk605*	23.2%
SOCG_01918	*SZOCTO_07208*	*tdk606*	26.0%

c

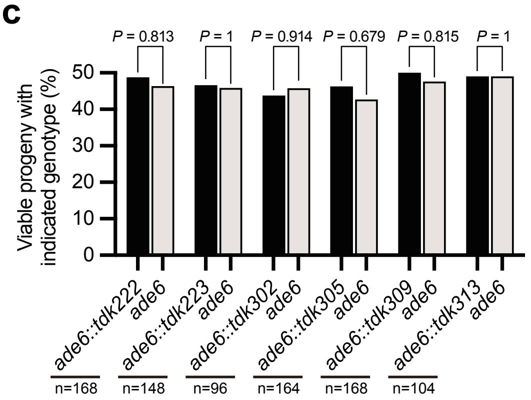

b

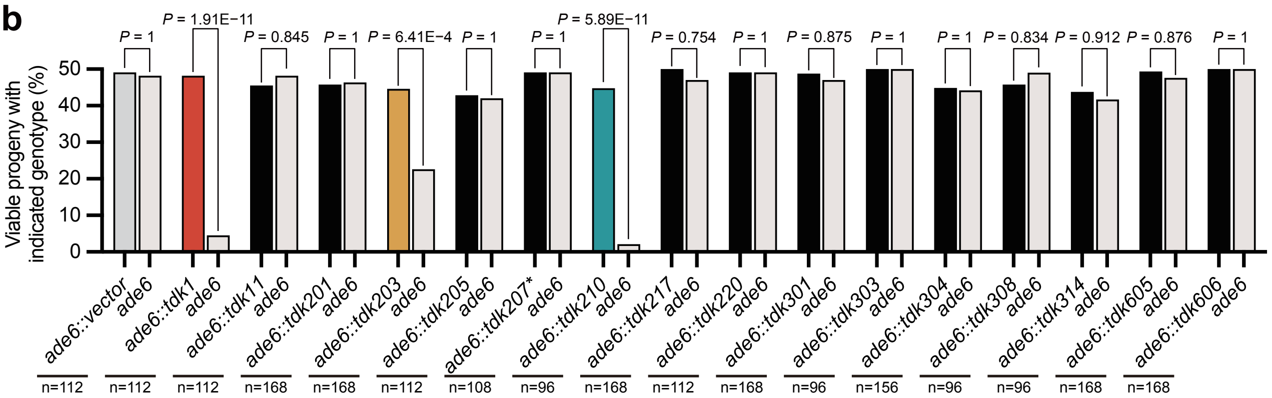

Supplementary Fig. 1: Identification of active KMDs among *tdk* homologs across fission yeasts.

a, Pairwise amino acid identity of 15 homologs with Tdk1 (calculated using EMBL-EBI Needle[54]), with previous RefSeq systematic ID, updated systematic ID, and new gene symbols.

b,**c** Tetrad analyses of 15 previously identified *tdk* homologs (**b**) and six additional *tdk* genes from *S. cryophilus* and *S. osmophilus* (**c**), revealing *tdk210* and *tdk203*—and no other homologs—as active KMDs. Each gene was controlled by *tdk1* promoter (*P.tdk1*) and terminator (*T.tdk1*), and integrated at the *ade6* locus in a *tdk1Δ tdk11Δ* background. *P* values (exact binomial test) compare observed viable progeny counts of the two genotypes to the expected 1:1 Mendelian segregation ratio. n, total progeny analyzed.

bioRxiv preprint doi: https://doi.org/10.64898/2025.12.28.696746; this version posted December 29, 2025. The copyright holder for this preprint (which was not certified by peer review) is the author/funder, who has granted bioRxiv a license to display the preprint in perpetuity. It is made available under aCC-BY 4.0 International license.

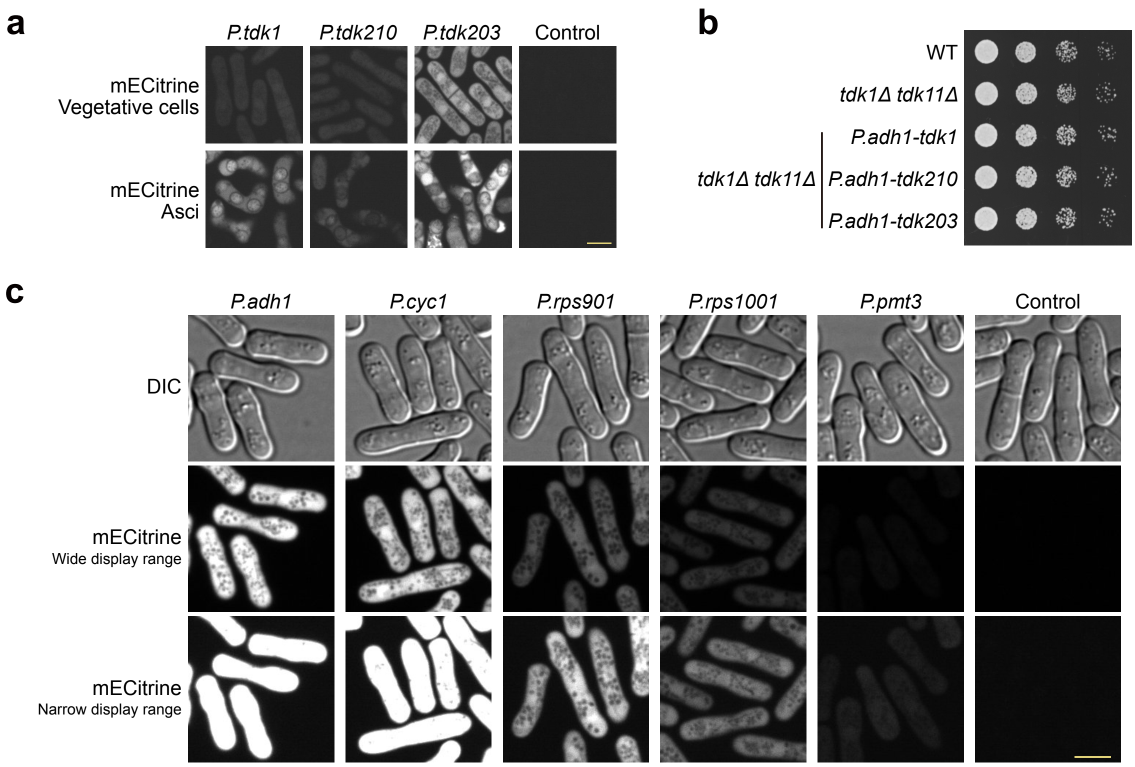

Supplementary Fig. 2: Transcriptional regulation of *tdk* drivers.

a, Fluorescence micrographs of vegetative cells and asci, showing meiotic upregulation of mECitrine expression driven by *P.tdk1* but not by *P.tdk210* or *P.tdk203*. Strains carried the indicated *ade6*-integrated *promoter-mECitrine* constructs in a *tdk1Δ tdk11Δ* background. Asci were obtained by crossing each strain with a *tdk1Δ tdk11Δ* strain. Scale bar, 5 μm.

b, Spot assays showing no vegetative toxicity of *tdk1*, *tdk210*, or *tdk203* expressed from the strong constitutive promoter *P.adh1* on the rich medium (YES).

c, Fluorescence micrographs illustrating the relative expression strengths of the constitutive promoters *P.adh1*, *P.cyc1*, *P.rps901*, *P.rps1001*, and *P.pmt3* used in Fig. 2c. Scale bar, 5 μm.

bioRxiv preprint doi: https://doi.org/10.64898/2025.12.28.696746; this version posted December 29, 2025. The copyright holder for this preprint (which was not certified by peer review) is the author/funder, who has granted bioRxiv a license to display the preprint in perpetuity. It is made available under aCC-BY 4.0 International license.

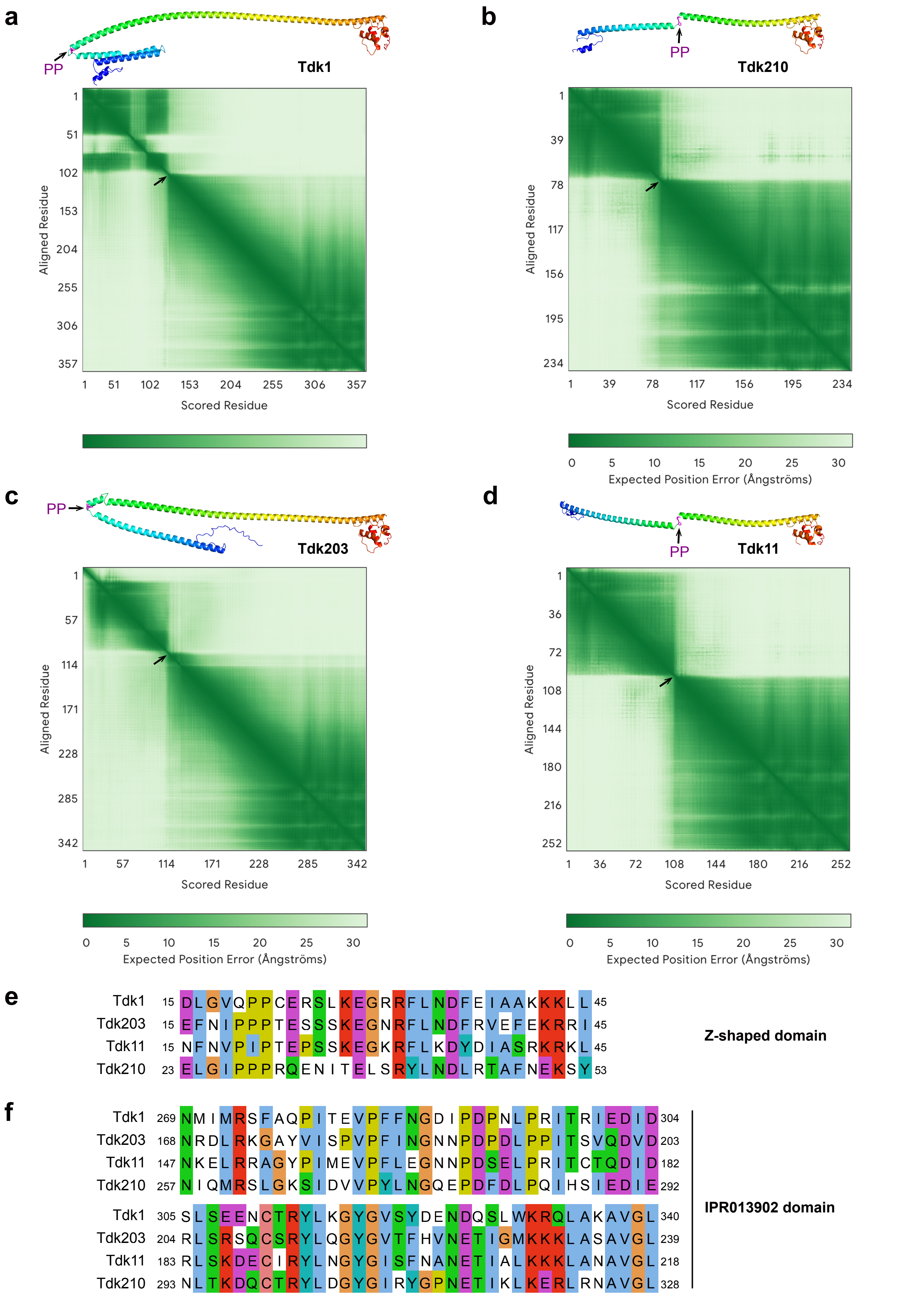

bioRxiv preprint doi: https://doi.org/10.64898/2025.12.28.696746; this version posted December 29, 2025. The copyright holder for this preprint (which was not certified by peer review) is the author/funder, who has granted bioRxiv a license to display the preprint in perpetuity. It is made available under aCC-BY 4.0 International license.

Supplementary Fig. 3: Structural predictions and conservation of Tdk driver proteins.

a–d, AlphaFold 3[26]-predicted monomeric structures of Tdk1 (**a**), Tdk210 (**b**), Tdk203 (**c**), and Tdk11 (**d**), with corresponding predicted aligned error (PAE) plots shown on the right. Structures are rainbow-colored from N- to C-terminus; the conserved PP motif is highlighted in magenta (stick representation) and indicated by a black arrow in the PAE plots.

e,**f** Sequence alignment of the conserved regions among Tdk1, Tdk203, Tdk210, and Tdk11: the N-terminal Z-shaped domain (**e**) and the IPR013902 domain (**f**).

bioRxiv preprint doi: https://doi.org/10.64898/2025.12.28.696746; this version posted December 29, 2025. The copyright holder for this preprint (which was not certified by peer review) is the author/funder, who has granted bioRxiv a license to display the preprint in perpetuity. It is made available under aCC-BY 4.0 International license.

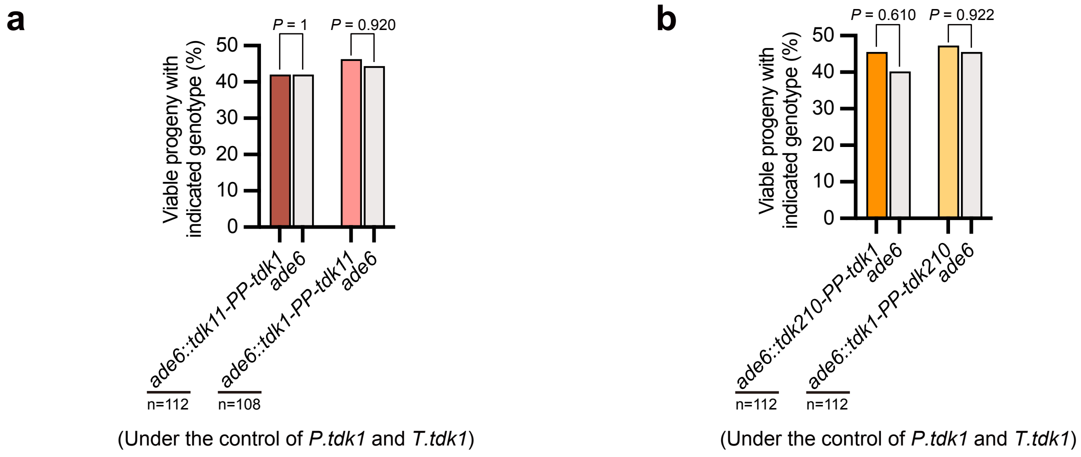

Supplementary Fig. 4: *tdk11–tdk1* and *tdk210–tdk1* chimeras lack drive activity. **a**,**b** Tetrad analyses of *tdk11–tdk1* (**a**) and *tdk210–tdk1* (**b**) chimeras (*tdk11-PP-tdk1*, *tdk1-PP-tdk11*, *tdk210-PP-tdk1*, *tdk1-PP-tdk210*) showing no drive activity. All constructs were under the control of *P.tdk1* and *P.tdk1*. *P* values (exact binomial test) compare progeny viability of the two genotypes within each cross. n, total progeny analyzed.

bioRxiv preprint doi: https://doi.org/10.64898/2025.12.28.696746; this version posted December 29, 2025. The copyright holder for this preprint (which was not certified by peer review) is the author/funder, who has granted bioRxiv a license to display the preprint in perpetuity. It is made available under aCC-BY 4.0 International license.

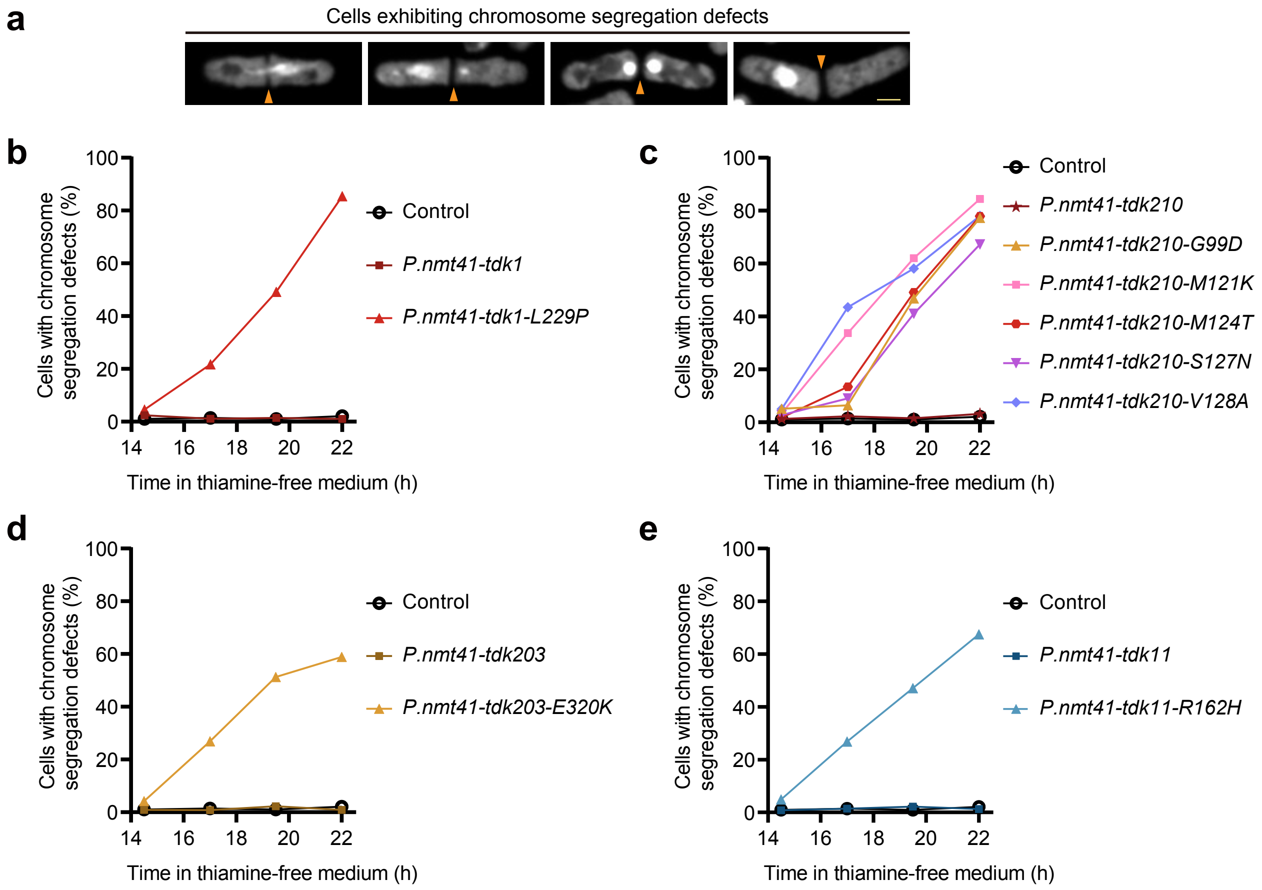

Supplementary Fig. 5: Self-killing *tdk* mutants disrupt mitotic chromosome segregation.

a, Representative micrographs of cells with chromosome segregation defects upon expression of self-killing Tdk variants. Nuclei were stained with SYTOX Green nucleic acid stain; orange arrows indicate septa. Scale bar, 2 μm.

b–e, Quantification of cells exhibiting chromosome segregation defects at the indicated time points following thiamine removal, which induces expression from the thiamine-repressible *P.nmt41* promoter[51]. Shown are wild-type and self-killing *tdk* variants whose expression was induced: *tdk1* and *tdk1-L229P* (**b**); *tdk210* and *tdk210-G99D*/*M121K*/*M124T*/*S127N*/*V128A* (**c**); *tdk203* and *tdk203-E320K* (**d**); *tdk11* and *tdk11-R162H* (**e**).

bioRxiv preprint doi: https://doi.org/10.64898/2025.12.28.696746; this version posted December 29, 2025. The copyright holder for this preprint (which was not certified by peer review) is the author/funder, who has granted bioRxiv a license to display the preprint in perpetuity. It is made available under aCC-BY 4.0 International license.

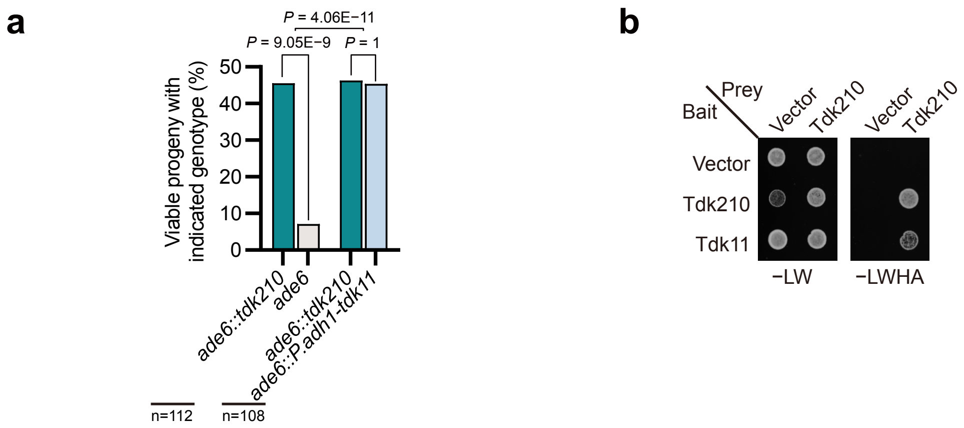

Supplementary Fig. 6: Tdk11 confers resistance to *tdk210*-mediated killing and interacts with Tdk210.

a, Tetrad analyses of *ade6::P.adh1-tdk11* × *ade6::tdk210* and *ade6* × *ade6::tdk210* (control) crosses, showing full resistance of *P.adh1-tdk11* to *tdk210*-mediated killing. *P* values within individual crosses were calculated using the exact binomial test; The *P* value between crosses were calculated using Fisher's exact test. n, total progeny analyzed.

b, Y2H assays demonstrating a physical interaction between Tdk11 and Tdk210.

bioRxiv preprint doi: https://doi.org/10.64898/2025.12.28.696746; this version posted December 29, 2025. The copyright holder for this preprint (which was not certified by peer review) is the author/funder, who has granted bioRxiv a license to display the preprint in perpetuity. It is made available under aCC-BY 4.0 International license.

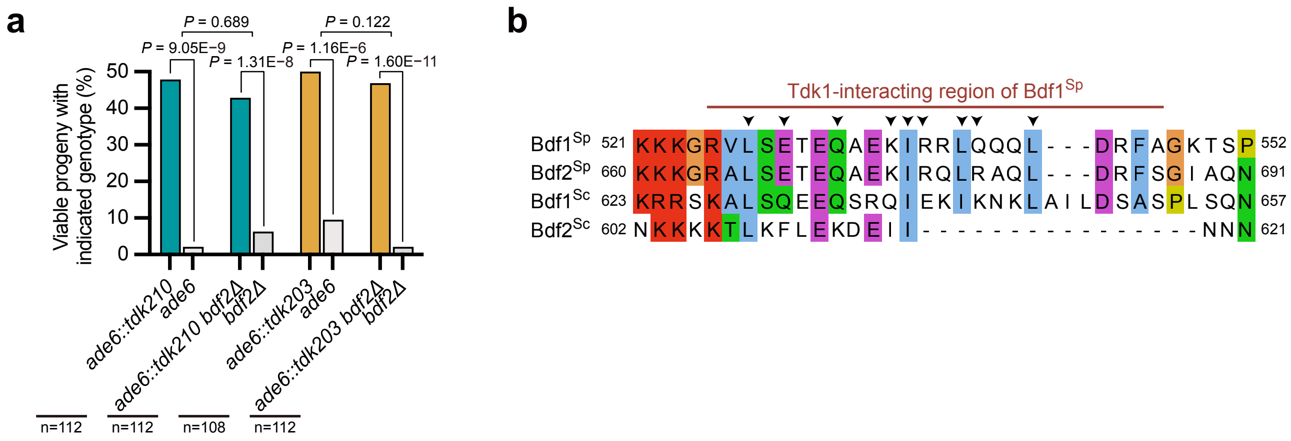

Supplementary Fig. 7: *tdk210* and *tdk203* drive independently of Bdf2.

a, Tetrad analyses of *ade6::tdk210* × *ade6* and *ade6::tdk203* × *ade6* in *bdf2+* and *bdf2Δ* backgrounds, showing full drive activity of *tdk210* and *tdk203* in the absence of *bdf2*. *P* values within individual crosses were calculated using the exact binomial test; *P* values between crosses were calculated using Fisher's exact test. n, total progeny analyzed.

b, Sequence alignment of the Tdk1-binding regions of *S. cerevisiae* Bdf1 (Bdf1^{Sc}) and Bdf2 (Bdf2^{Sc}) with their orthologs in *S. pombe* (Bdf2^{Sp} and Bdf1^{Sp}), showing that the corresponding regions in Bdf1^{Sc} and Bdf2^{Sc} exhibit low sequence conservation compared to those in *S. pombe*. Arrows indicate residues in Bdf1^{Sp} that directly interact with Tdk1[21].

bioRxiv preprint doi: https://doi.org/10.64898/2025.12.28.696746; this version posted December 29, 2025. The copyright holder for this preprint (which was not certified by peer review) is the author/funder, who has granted bioRxiv a license to display the preprint in perpetuity. It is made available under aCC-BY 4.0 International license.

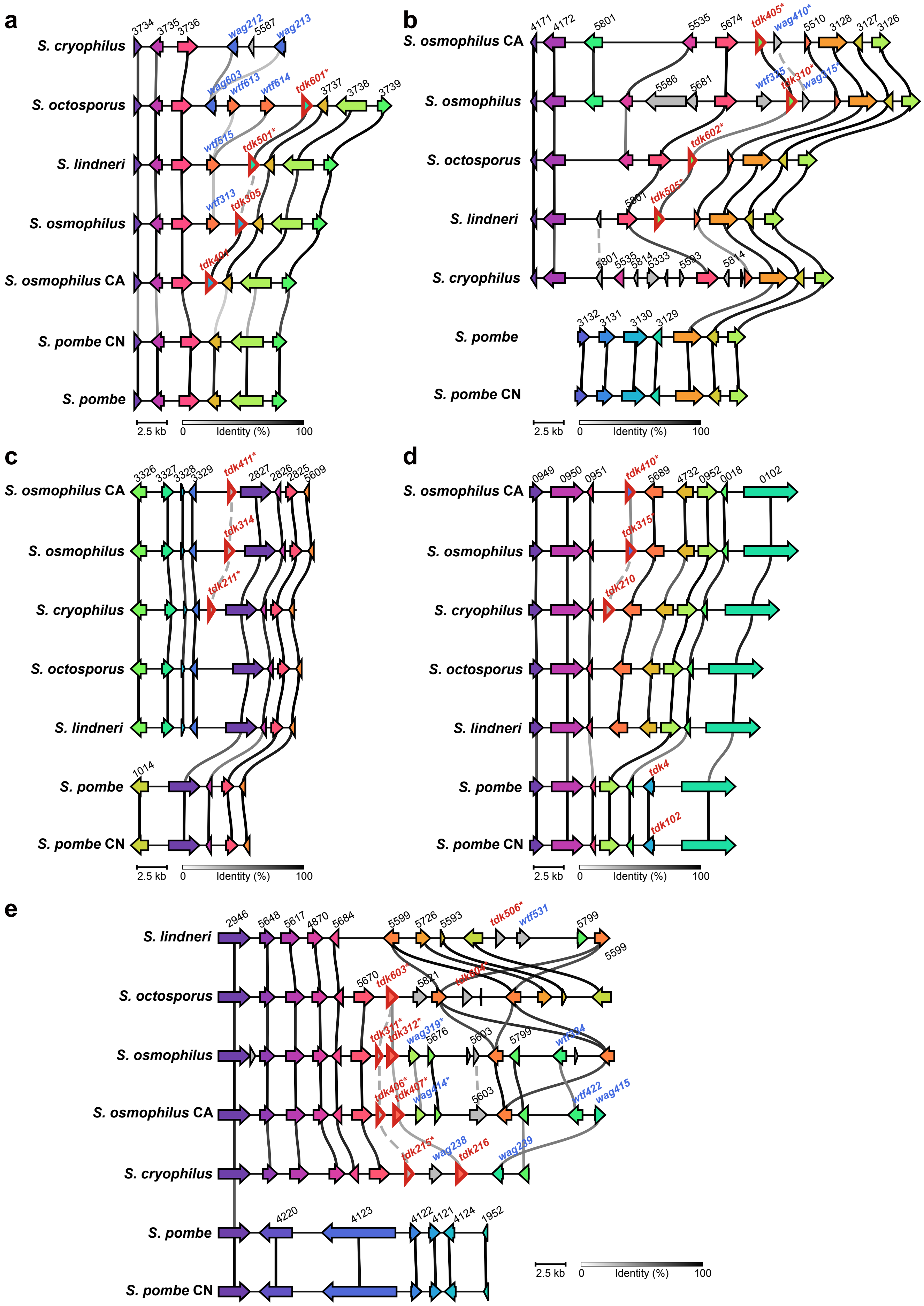

1

bioRxiv preprint doi: https://doi.org/10.64898/2025.12.28.696746; this version posted December 29, 2025. The copyright holder for this preprint (which was not certified by peer review) is the author/funder, who has granted bioRxiv a license to display the preprint in perpetuity. It is made available under aCC-BY 4.0 International license.

Supplementary Fig. 8: Cross-species synteny of *tdk* gene loci in the *Schizosaccharomyces* genus.

a–e, Synteny analysis identified five cross-species synteny sets of *tdk* loci: two within the *osmo–lind–octo* group (*S. osmophilus*, *S. osmophilus* CA, *S. lindneri*, and *S. octosporus*) (**a**,**b**); two shared among *S. cryophilus*, *S. osmophilus*, and *S. osmophilus* CA (**c**,**d**); and one conserved in *S. cryophilus*, *S. osmophilus*, *S. osmophilus* CA, and *S. octosporus* (**e**). Four-digit numbers denote synteny group IDs from the *Schizosaccharomyces* Orthogroup (SOG) resource (https://fsnibs10.github.io/SOG/). *wtf* and *wag* genes, frequently found flanking *tdk* loci, are also labeled. Synteny plots were generated using Clinker[45]. Conserved genomic blocks are connected by solid lines, with line color indicating sequence identity. Only homologous regions sharing ≥ 30% sequence identity are linked by solid lines; connections below this threshold were manually added as gray dashed lines.

bioRxiv preprint doi: https://doi.org/10.64898/2025.12.28.696746; this version posted December 29, 2025. The copyright holder for this preprint (which was not certified by peer review) is the author/funder, who has granted bioRxiv a license to display the preprint in perpetuity. It is made available under aCC-BY 4.0 International license.

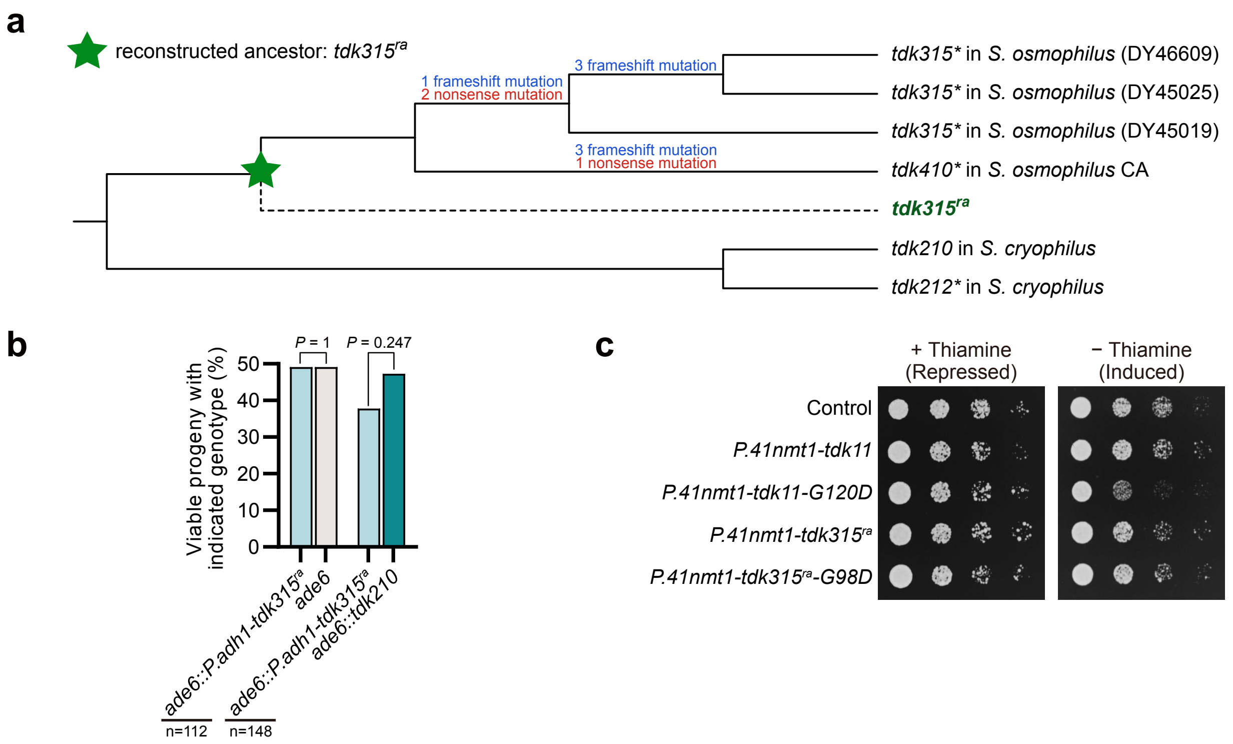

Supplementary Fig. 9: The ancestral state of *tdk315* is likely a resistant allele.

a, Schematic of ancestral sequence reconstruction of *tdk315^{ra}*. *S. osmophilus* CA and natural isolates of *S. osmophilus* carry distinct inactivating mutations (frameshift and nonsense mutations), consistent with degenerative evolution. *tdk210* and *tdk212** from *S. cryophilus* were used as outgroups for ancestral inference (see Methods). The reconstructed ancestral sequence is designated *tdk315^{ra}*.

b, Tetrad analyses of *ade6::P.adh1-tdk315^{ra}* × *ade6* and *ade6::P.adh1-tdk315^{ra}* × *ade6::tdk210* crosses, showing that *P.adh1-tdk315^{ra}* lacks drive activity but confers strong resistance to *tdk210*-mediated killing. *P* values (exact binomial test) compare progeny viability of the two genotypes within each cross. n, total progeny analyzed.

c, Spot assays showing toxicity of *tdk11-G120D* but not *tdk315^{ra}-G98D* when expressed from the *P.nmt41* promoter. The G120D substitution in *tdk11* and G98D in *tdk315^{ra}* correspond to the self-killing G99D mutation in *tdk210*.

bioRxiv preprint doi: https://doi.org/10.64898/2025.12.28.696746; this version posted December 29, 2025. The copyright holder for this preprint (which was not certified by peer review) is the author/funder, who has granted bioRxiv a license to display the preprint in perpetuity. It is made available under aCC-BY 4.0 International license.

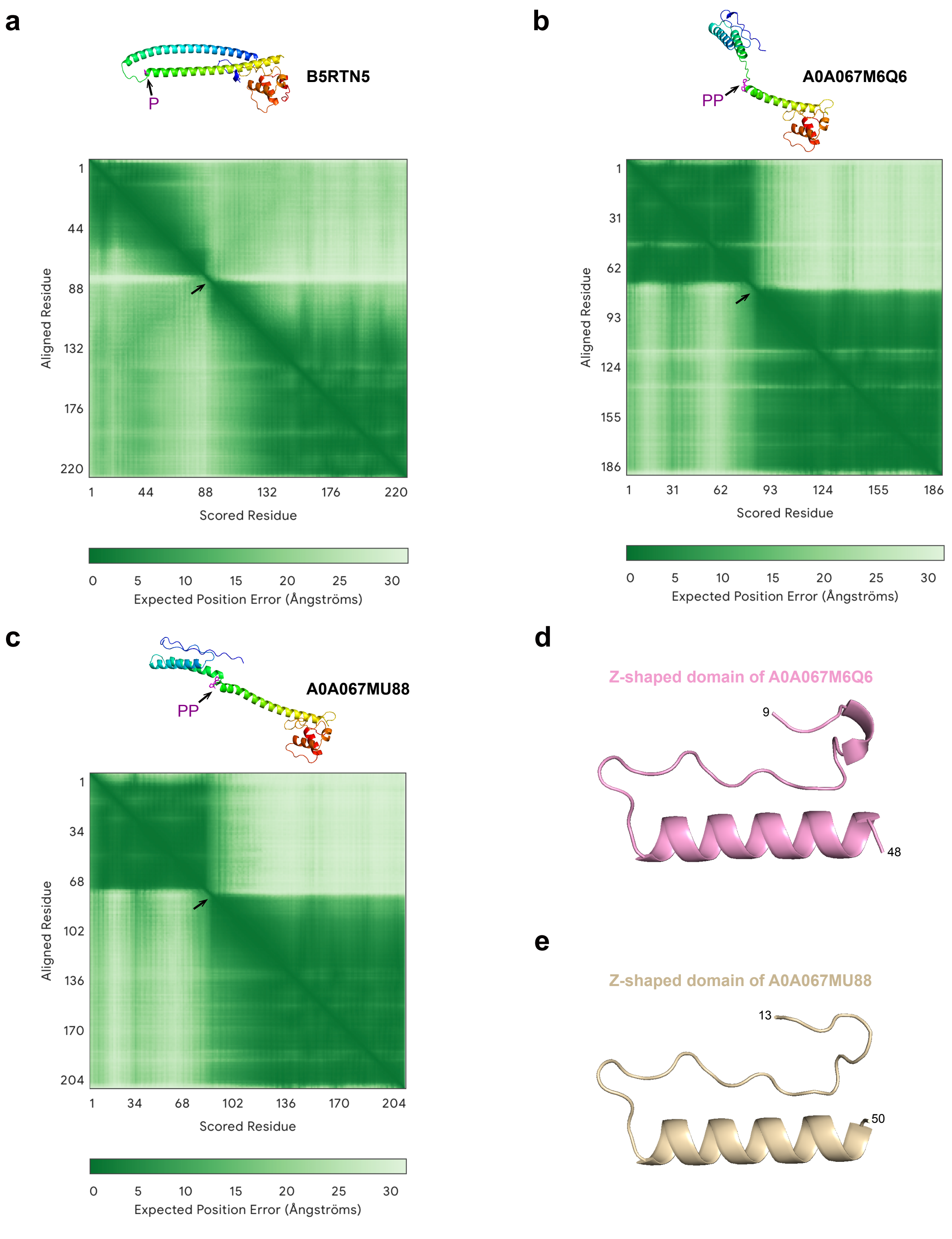

Supplementary Fig. 10: Structural predictions and similarity of non-*Schizosaccharomyces* Tdk homologs to *Schizosaccharomyces* Tdk driver proteins. **a–c**, AlphaFold 3[26]-predicted monomeric structures of B5RTN5 (**a**), A0A067M6Q6 (**b**), and A0A067MU88 (**c**), with PAE plots shown on the right. Structures are rainbow-colored from N- to C-terminus; the conserved PP motif is highlighted in magenta (stick

bioRxiv preprint doi: https://doi.org/10.64898/2025.12.28.696746; this version posted December 29, 2025. The copyright holder for this preprint (which was not certified by peer review) is the author/funder, who has granted bioRxiv a license to display the preprint in perpetuity. It is made available under aCC-BY 4.0 International license.

representation) and indicated by a black arrow in the PAE plots. B5RTN5 contains a single proline at the position corresponding to the PP motif of *Schizosaccharomyces* Tdk driver proteins.

d,**e**, Predicted N-terminal Z-shaped structure of A0A067M6Q6 (**d**) and A0A067MU88 (**e**), similar to that of *Schizosaccharomyces* Tdk driver proteins, despite the failure of structural superposition.

bioRxiv preprint doi: https://doi.org/10.64898/2025.12.28.696746; this version posted December 29, 2025. The copyright holder for this preprint (which was not certified by peer review) is the author/funder, who has granted bioRxiv a license to display the preprint in perpetuity. It is made available under aCC-BY 4.0 International license.

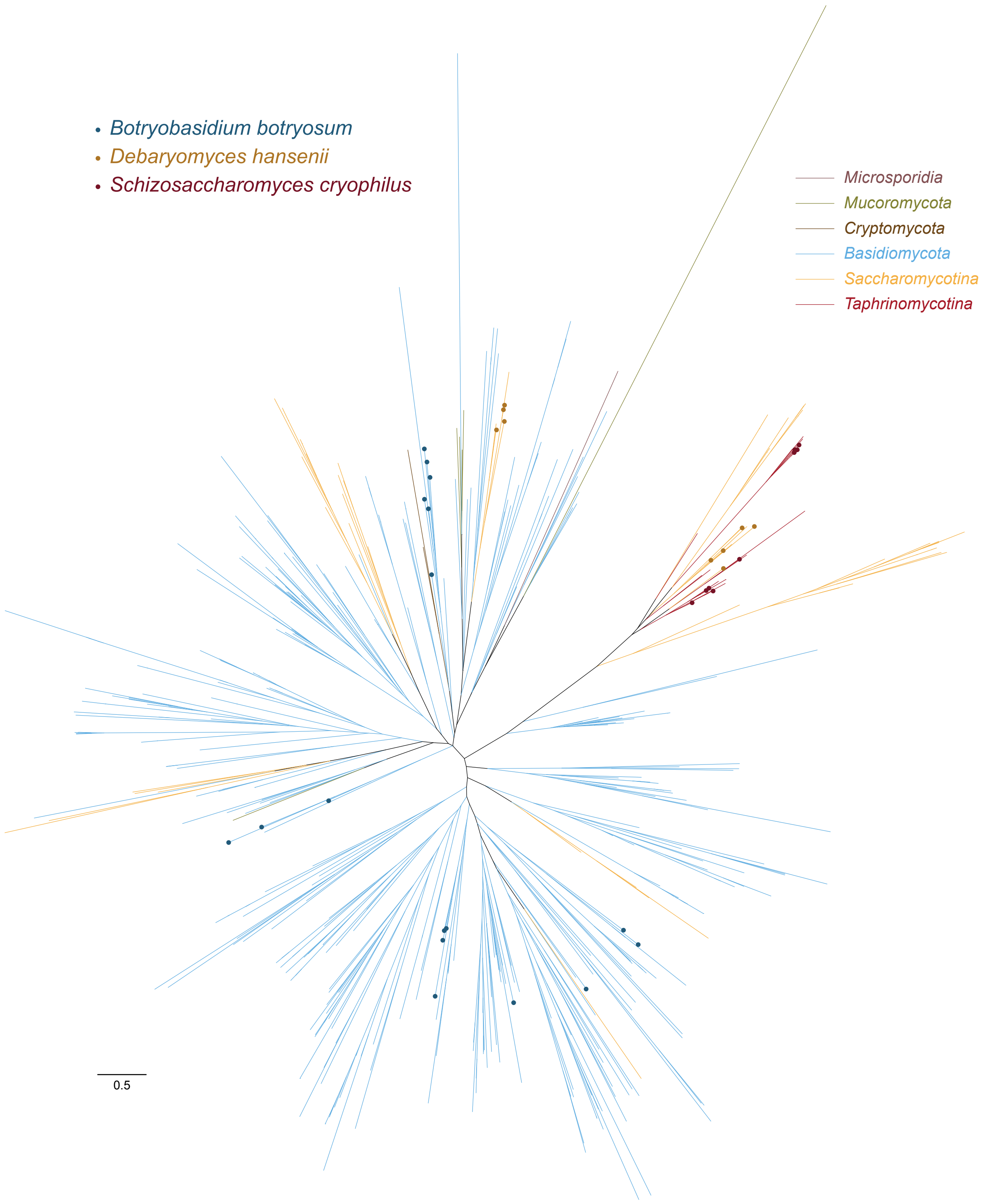

Supplementary Fig. 11: *tdk* homologs in *Debaryomyces hansenii* and *Botryobasidium botryosum* exhibit greater sequence divergence than those in *Schizosaccharomyces cryophilus*.

Maximum-likelihood phylogenetic tree of fungal IPR013902-containing proteins (Fig. 7c), highlighting 9 *D. hansenii*, 17 *B. botryosum*, and 11 *S. cryophilus* homologs. The

bioRxiv preprint doi: https://doi.org/10.64898/2025.12.28.696746; this version posted December 29, 2025. The copyright holder for this preprint (which was not certified by peer review) is the author/funder, who has granted bioRxiv a license to display the preprint in perpetuity. It is made available under aCC-BY 4.0 International license.

tree file is provided in Supplementary Data 3. Scale bar: 0.5 substitutions per site.
